# A Study of Carbon Emission Efficiency in Chinese Provinces Based on a Three-Stage SBM-Undesirable Model and an LSTM Model

**DOI:** 10.3390/ijerph19095395

**Published:** 2022-04-28

**Authors:** Huayong Niu, Zhishuo Zhang, Yao Xiao, Manting Luo, Yumeng Chen

**Affiliations:** 1 International Business School, Beijing Foreign Studies University, Beijing 100089, China; niuhuayong@bfsu.edu.cn (H.N.); xiaoyaoibs@bfsu.edu.cn (Y.X.); manting@bfsu.edu.cn (M.L.); 2 Liaoning Banking and Insurance Regulatory Bureau, Shenyang 110013, China; chenyumeng321@126.com

**Keywords:** carbon emissions, carbon emission efficiency, three-stage DEA, SBM-undesirable, SFA model, LSTM model

## Abstract

As a major carbon-emitting country, there is an urgent need for China to reduce carbon emissions. Studying the carbon emission efficiency of each province helps us to learn about the characteristics and evolution of regional carbon emissions, which is important for proposing effective and targeted measures to achieve the carbon peaking and carbon neutrality goals. This paper measures the carbon emission efficiency of 30 Chinese provinces from 2006 to 2019 based on a three-stage SBM-undesirable model and explores external drivers using stochastic frontier models. The results of the SBM-undesirable model show that the inter-provincial carbon emission efficiency is unevenly distributed and shows a big difference. From the results of the stochastic frontier model analysis, external drivers such as the intensity of finance in environmental protection, the level of economic development, the industrial structure, the level of urbanization, the degree of openness and the level of science as well as technology innovation all have an impact on the emission efficiency. In terms of LSTM model prediction, the model shows an excellent fitting effect, which provides a possible path for carbon emission efficiency prediction. Finally, based on the empirical results and the actual situation of each province in China, this paper proposes relevant feasible suggestions.

## 1. Introduction

With the development of economy and technology, environmental problems such as global warming and land desertification have emerged one after another. In addition, many unstable factors in the international community have exacerbated environmental problems, which have gradually become a hot issue in the world. Therefore, as an important factor in environmental issues, climate change has become one of the most urgent environmental issues facing the international community and governments. In September 2020, President Xi announced to the world at the general debate of the 75th United Nations General Assembly that China would strive to achieve carbon peaking by 2030 and carbon neutrality by 2060. On 15 March 2021, President Xi presided over the ninth meeting of the Central Finance and Economics Commission and delivered an important speech, emphasizing the need to integrate carbon peaking and carbon neutrality into the overall layout of ecological civilization construction, so as to achieve goals of peak carbon by 2030 and carbon neutrality by 2060 on schedule. The introduction of the “double carbon” target reflects China’s importance, and China has elevated the achievement of the “double carbon” target to a national strategy. At the same time, along with the increasing energy consumption and environmental pollution caused by economic growth, people have gradually realized the importance of a low-carbon economy. Considering the development of different regions, the first and foremost aspect of the government’s development of a low-carbon economy is to carry out regional energy conservation and emission reduction efforts to improve the efficiency of regional carbon emissions.

As countries pay more attention to carbon emission reduction, how to measure the carbon emission efficiency has gradually become a popular area of academic research. From the definition of carbon emission efficiency, there is no authoritative definition in academia yet, and it is mainly divided into single-factor and full-factor perspectives of carbon emission efficiency. From the single-factor perspective, the main measurement indicators are carbon dioxide emissions per unit of GDP per capita or carbon dioxide emissions per unit of GDP [1,2] and carbon emissions from energy consumption in terms of tons of standard coal [3]. More scholars now use carbon productivity to represent single-factor carbon emission efficiency, which is defined as the economic output created per unit of carbon emissions, usually expressed as GDP per unit of carbon emissions [4,5,6]. From the perspective of total-factor efficiency, the main indicator is the comprehensive efficiency of carbon emissions [7,8] or the total-factor efficiency of carbon emissions [9], which reflects the characteristic of total-factor efficiency that combined with economic development and other indicators, and it can measure the efficiency of carbon emissions more accurately and comprehensively.

There are two main methods to measure total-factor carbon emission efficiency, SFA and DEA, with the DEA being the most popular. Lei and Yang [10] used the SFA model and added seven variables that have an influence on carbon emission efficiency to the model to measure the carbon emission efficiency of 30 Chinese provinces from 1996 to 2011. Although some measure efficiency using the SFA method, more scholars use DEA to measure it. This is mainly because the DEA method does not require the setting of a specific functional form, thus avoiding the structural bias of the SFA method due to the mis-setting of the production function. Tyteca [11] used the CCR model in DEA to evaluate environmental performance from the perspective of inputs and outputs. Ma et al. [12] used the BCC model to measure the carbon emission performance of the logistics industry in 30 Chinese provinces. Moutinho et al. [13] used CCR and BCC models to measure the economic and environmental efficiency of 26 different European countries from 2001 to 2012. Considering the inability of the traditional CCR and BCC models to measure panel data, the DEA window analysis model is proposed. Iftikhar et al. [14] used the DEA window analysis model to assess the energy and CO2 emission efficiency of major economies. Yang et al. [15] also used DEA window analysis to evaluate urban sustainability in Taiwan. Sueyoshi et al. [16] evaluated the environmental performance of provinces in China from 2003 to 2014 by using a DEA window analysis model. Vlontzos et al. [17] similarly used a DEA window analysis model to assess the efficiency of greenhouse gas emissions in the process of agricultural production in EU countries. Furthermore, there is literature that considered the effect of external drivers with stochastic noise, and then measured carbon emission efficiency through a three-stage DEA [18]. This method used SFA proposed by Aigner et al. [19] in addition to the DEA model to calculate the effects of external drivers and random errors on carbon emission efficiency, so that the calculation of emission efficiency can be more accurate. Hua et al. [20] evaluated the provincial carbon dioxide emission performance in China by using a three-stage DEA and linear function transformation method. Zhang et al. [21] studied the carbon emission efficiency of the construction industry in China based on a three-stage DEA model. While radial DEA models are mostly used in traditional DEA, nonradial SBM can measure efficiency values more accurately, which can be better applied in the carbon emission efficiency measurement. Iqbal et al. [22] used an undesirable output SBM model to measure energy consumption, carbon emissions, and economic environmental efficiency in the top 20 industrial countries from 2013 to 2017. Anser et al. [23] studied environmental efficiency using a comparative radial DEA and nonradial DEA, and the results showed that in the measurement of environmental performance analysis, the nonradial DEA had higher discriminability than radial DEA. Based on the aforementioned studies, we fully integrate the three-stage DEA and the SBM-undesirable models to measure carbon emission efficiency in 30 provinces of China.

Most studies stopped at the exploration of carbon emission efficiency measurement and influencing factors, and there is a lack of research on carbon emission efficiency prediction. In all fields of forecasting methods, there are few studies combining DEA with neural network models. The idea of combining DEA and neural networks was first proposed by Athanassopoulos and Curram [24], using DEA as a preprocessing method for screening training cases and ANNs as a tool for learning nonlinear forecasting models. In particular, in the field of carbon emissions, some scholars used neural network models in their predictions [25,26], but these models were not combined with DEA. Some studies compared neural network models with econometric models in terms of prediction accuracy. Zhou and Kuang [27] found that neural network models, especially the LSTM model, have higher accuracy in prediction than the traditional VAR model. Econometric models are commonly used in forecasting economics research, and such models are mainly based on linear relationships. While neural network models, which use gradient descent to search for the optimal global solution as well as have activation function and feedback mechanism, can better capture the nonlinear relationships, so they have obvious advantages in dealing with nonlinear, discontinuous and high-dimensional data [28]. Ouyang et al. [29] compared the prediction effectiveness of LSTM model with four models, namely, multilayer perceptron, support vector machine, K-nearest neighbor and GARCH, and the empirical results showed that LSTM model has higher prediction accuracy. It can effectively predict the dynamic trends of financial time series data in the long and short terms.

The important scientific contributions of this paper include the following: (1) Most of the carbon emission efficiency measurement methods are the undesirable SBM model and the traditional three-stage DEA model. In this paper, considering that the two models have different advantages, we combine the two to measure the carbon emission efficiency, which expands the modeling methods in this field. (2) This paper uses the SFA model regression to verify that six external drivers, including the intensity of finance in environmental protection, level of economic development, industrial structure, level of urbanization, degree of openness and level of science and technology innovation, have significant effects on the value of carbon emission efficiency, and proposes relevant policy recommendations based on the regression results. (3) In this paper, we use the LSTM model to forecast the labor force, capital stock, total energy consumption, gross regional product and carbon dioxide emissions in the next five years, and then measure the carbon emission efficiency of 30 provinces in China from 2020 to 2024. The empirical results show that the model has good prediction effect and can effectively predict the carbon emission efficiency. (4) This paper combines the data envelopment analysis method with a neural network model, which is a methodological expansion in the field of performance evaluation and prediction, and achieves good empirical results with certain generalization ability, further enriching the research ideas in this field.

In this paper, we construct an input–output evaluation index system containing nonexpectation indicators in a total-factor perspective, measure carbon emission efficiency values using a three-stage SBM-undesirable model, explore the external drivers of carbon emission efficiency through SFA regression, and finally forecast the carbon emission efficiency of thirty Chinese provinces in the next five years using an LSTM model. Based on the empirical study, this paper analyzes the current state of carbon emission in 30 provinces of China from 2006 to 2019, explores carbon emission reduction path in China, and then offers targeted policy recommendations to promote low-carbon development. The structure of this paper is as follows: in the second part, we introduce the basic models and methods involved in this study; in the third part, we design a system of evaluation and select some important external drivers; in the fourth part, we conduct carbon emission efficiency measurement, regression analysis of external drivers and prediction based on the above designed system and selected model. We also conduct an empirical analysis and discussion of the results; in the fifth part, we summarize main research findings and put forward corresponding recommendations based on these findings for the reference of relevant institutions.

## 2. Description of the Methodology

### 2.1. SBM-Undesirable Model

In 1978, Charnes, Cooper, and Rhodes created the first theoretical approach of DEA, the CCR model, which is named after the initials of these three individuals’ last names. They extended the concept of single-input, single-output engineering efficiency to multiple-input, multiple-output relative efficiency evaluation [30]. The CCR model measures the relative efficiency of the decision-making unit (DMU) under the assumption of constant returns to scale. However, in practice, returns to scale are generally variable. Banker et al. [31] then proposed for the first time to evaluate the relative efficiency of a DMU using the variable returns to scale as a criterion, which is the BCC model.

Since the DEA-CCR and BCC models cannot measure the full range of slack variables, there are defects in efficiency evaluation. For improvement, Tone [32] proposed the SBM model, which took into account the slack of input–output and made the efficiency measurement more accurate. To solve the problem that the SBM model cannot measure the efficiency of DMUs with undesirable outputs, Tone [33] then proposed an SBM model that considers undesirable outputs. The mathematical programming formula is:

(1) $$\rho =\min\frac{1-\frac{1}{m}\sum_{i=1}^{m}\frac{s_{i}^{-}}{x_{i0}}}{1+\frac{1}{s_{1}+s_{2}}(\sum_{r=1}^{s_{1}}\frac{s_{r}^{g}}{y_{r0}^{g}}+\sum_{r=1}^{s_{2}}\frac{s_{r}^{b}}{y_{r0}^{b}})}\;\begin{array}{c} \;s.t.\;\{\begin{array}{c} x_{0}=X\lambda +s^{-}\\ y_{0}^{g}=Y^{g}\lambda -s^{g}\\ y_{0}^{b}=Y^{b}\lambda +s^{b}\\ s^{-}\geq 0,s^{g}\geq 0,s^{b}\geq 0,\lambda \geq 0 \end{array} \end{array}$$

Each DMU in the model consists of three variables, input, desired output and undesirable output, $x\in R^{m}$, $y^{g}\in R^{s_{1}}$ and $y^{b}\in R^{s_{2}}$, respectively. ρ is the objective function, which is strictly decreasing and satisfies 0≤ρ≤1. $s^{-}$, $s^{g}$, $s^{b}=0$ are the input slack variable, desirable output slack variable and undesirable output slack variable, respectively. For a particular DMU, it is efficient when and only when ρ=1 and $s^{-}=0$, $s^{g}=0$, $s^{b}=0$. Conversely, the DMU is relatively inefficient.

### 2.2. Three-Stage DEA Model

Fried et al. [18] proposed that the traditional DEA model did not remove the effects of managerial inefficiency, external drivers and random noise on the efficiency values, and they discussed how to introduce the above factors into the DEA model, which is the three-stage DEA model.

The three-stage DEA model solves the problem through three main stages. In the first stage, the traditional DEA model is used to evaluate the initial efficiency based on the original input–output data. In this paper, the SBM model, which considers undesirable outputs, is used in this stage to overcome the shortcomings of the traditional DEA model.

In the second stage, the main focus is on the SFA regression of slack variables, composed of management inefficiency, external drivers and random noise, and the slack variables are decomposed into the above three effects. If the first stage is input oriented, then the decomposition of the input slack variables is required. Next, we adjust the initial input variables according to the results, and the input-oriented SFA regression function is:

(2) $$\begin{array}{l} S_{ni}=f(Z_{i};\beta_{n})+\nu_{ni}+\mu_{ni}\\ i=1,2,\cdots ,I;n=1,2,\cdots ,N \end{array}$$

In Equation (2), $S_{ni}$ refers to the nth slack value of input of DMU*_i_*; *Z_i_* denotes the external driver variable, $\beta_{n}$ is the coefficient of the environment variable; $\nu_{ni}+\mu_{ni}$ refers to the mixed error term, $\nu_{ni}$ denotes random noise, and $\mu_{ni}$ denotes management inefficiency.

The purpose of the SFA regression is to remove the effects of external drivers and random noise on efficiency, adjusting all DMUs to the same external environment with the following adjustment formula:

(3) $$\begin{array}{l} X_{ni}^{A}=X_{ni}+[\max(f(Z_{i};{\overset{\wedge }{\beta }}_{n}))\\ -f(Z_{i};{\overset{\wedge }{\beta }}_{n})]+[\max(\nu_{ni})-\nu_{ni}]\\ i=1,2,\cdots ,I;n=1,2,\cdots ,N \end{array}$$

where $X_{ni}^{A}$ denotes adjusted input value; $X_{ni}$ is the input value before adjustment; $\left[\max(f(Z_{i};{\overset{\wedge }{\beta }}_{n}))-f(Z_{i};{\overset{\wedge }{\beta }}_{n})\right]$ refers to the adjustment for external drivers; and $\left[\max(\nu_{ni})-\nu_{ni}\right]$ denotes the adjustment for random noise of DMU*_i_*.

In the third stage, the adjusted input–output are remeasured applying the SBM model considering undesirable outputs. By the above process, the efficiency values at this time have removed the influence of external drivers and random noise, so as to obtain relatively accurate carbon emission efficiency values.

### 2.3. LSTM Model

**Figure 1:**
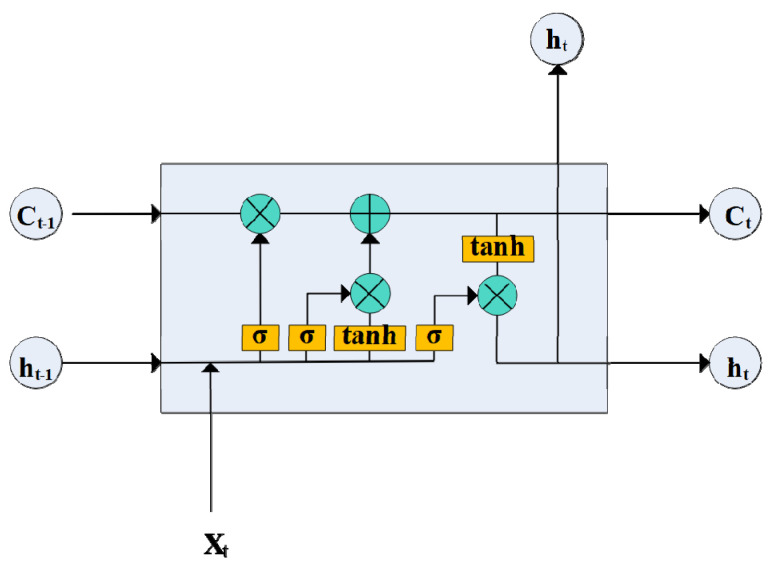
LSTM model core structure diagram.

The LSTM model, first proposed by Hochreiter and Schmidhuber [34], is a special form of recurrent neural network (RNN). The LSTM model is unique because it improves the RNN model by adding the gate structure, allowing the LSTM model to remove or add information to the nodes to change the state of the information flow. This feature also enables the LSTM model to learn better with regard to long-term information, and the core structure of the model is shown in Figure 1.

Specifically, h_t-1_ represents the result of the previous iteration, and X_t_ represents the new data vectors. Firstly, the LSTM model determines which messages to discard by passing through σ layer, known as the forgetting gate layer. This layer describes the situation of each information vector by outputting a number between 0 and 1, where 0 is not allowed to pass and 1 means pass. Subsequently, the σ layer, called the update gate layer, and the tanh layer determine which new information we will store in the unit node state, thus updating the learning outcome state C. Finally, the effective information is exported by the σ layer, called the output gate layer. This process can obtain a better prediction model by several iterations.

## 3. Construction of Carbon Emission Efficiency Evaluation Index System and Selection of External Drivers

### 3.1. Decision-Making Unit

The economic regions of China are divided into four major regions, namely, the eastern, the central, the western and the northeastern regions. Each region contains several provinces, and this paper takes the provinces of mainland China as the DMUs. Due to the missing data of Tibet, the eastern region contains ten provinces, the central region contains six provinces, the western region contains eleven provinces, and the northeastern region contains three provinces, each province is a decision unit, totaling thirty DMUs. Then, we analyze the carbon emission efficiency of each region and province in China according to the efficiency values of these DMUs.

### 3.2. Carbon Emission Efficiency Evaluation Index System

The input indicators in this paper include labor force, capital stock and total energy consumption. The desirable output indicator is regional GDP, and the undesirable output indicator is carbon dioxide emission. The carbon emission efficiency evaluation index system is shown in Table 1.

### 3.3. External Drivers

The external drivers are mainly selected from variables that have significant influence on carbon emission efficiency but are not endogenous. In this paper, six indicators are selected as external drivers, including the intensity of finance in environmental protection, the level of economic development, industrial structure, the level of urbanization, the degree of opening and the level of scientific and technological innovation, as listed in Table 2. The data were obtained from the China Statistical Yearbook, China Statistical Yearbook by province and the National Bureau of Statistics of China. The economic significance of each external driver is as follows:

1. Intensity of finance in environmental protection: Local government expenditures on environmental protection have an impact on carbon emission efficiency. In this paper, the ratio of local fiscal expenditure on environmental protection to local GDP is used to represent the intensity of local finance in environmental protection. The data of 2006 are missing, and considering that the fiscal expenditure ratio will not change too much in a short term, we use the ratio of 2007 to calculate the data of 2006.
2. Level of economic development: Many studies have found that there is a close relationship between the economic development and carbon emission efficiency. GDP per capita is an important indicator to measure the regional economic development level.
3. Industrial structure: The secondary industry is a high energy-consuming industry and emits more carbon dioxide than other industries during production. Therefore, we selected the ratio of the added value of secondary industry production to local GDP as an indicator to measure the industrial structure.
4. Level of urbanization: Urbanization brings an increase in energy consumption, and studies have shown that the increase in the level of urbanization has a significant impact on carbon dioxide emissions, thus affecting carbon emission efficiency. We use the ratio of urban population to the total local population to measure the level of urbanization.
5. Degree of openness: Opening up to the outside will introduce advanced technology, equipment, etc., which will help to further improve the energy efficiency and thus reduce carbon emissions. We use the ratio of total import and export volume to local GDP to measure the degree of openness.
6. Level of scientific and technological innovation: Scientific and technological progress will be conducive to improving production efficiency, promoting the use of clean energy, and thus improving the efficiency of carbon emissions. We use the ratio of R&D expenditure to local GDP to measure this indicator.

**Table 2:** Composition of external driver indicators.

Environmental Variables	Indicator Interpretation	Indicator Interpretation
Intensity of finance in environmental protection	Ratio of local fiscal expenditure on environmental protection to local GDP (%)	China Statistical Yearbook by province
Level of economic development	GDP per capita (Yuan/person)	China Statistical Yearbook
Industrial structure	Ratio of added value of secondary industry production to local GDP (%)	China Statistical Yearbook
Level of urbanization	Ratio of urban population to total local population (%)	China Statistical Yearbook
Level of openness	Ratio of total import and export to local GDP (%)	China National Bureau of Statistics
Level of scientific and technological innovation	Ratio of R&D expenditure to local GDP (%)	China Statistical Yearbook

## 4. Empirical Analysis

### 4.1. Analysis of the Initial Results of Carbon Emission Efficiency of Each Province Based on the First Stage SBM-Undesirable Model

**Figure 2:**
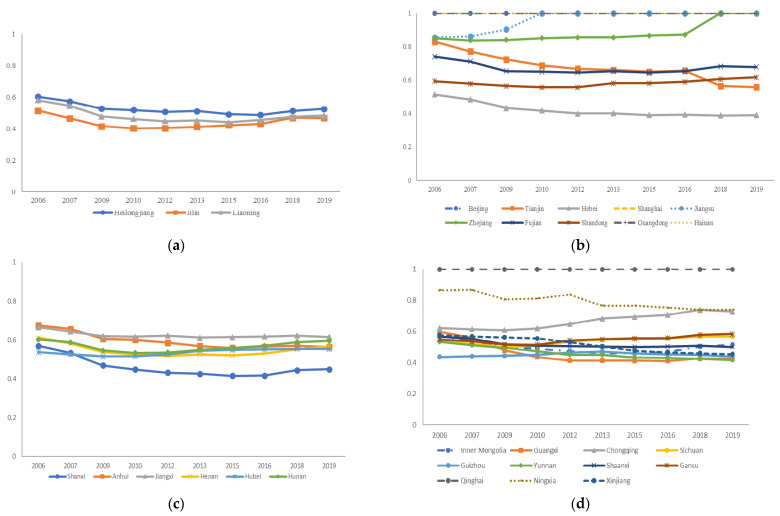
(**a**) Initial results of carbon emission efficiency in Northeastern China, 2006–2019; (**b**) initial results of carbon emission efficiency in Eastern China, 2006–2019; (**c**) initial results of carbon emission efficiency in Central China, 2006–2019; (**d**) initial results of carbon emission efficiency in Western China, 2006–2019. Note: To ensure the effect of the figure, the results of 2008, 2011, 2014, 2017 are not shown; please contact the author to obtain them if necessary.

In this paper, we used Matlab to construct the SBM-undesirable model, and the initial results of carbon emission efficiency of 30 Chinese provinces from 2006 to 2019 are shown in Figure 2.

**Figure 3:**
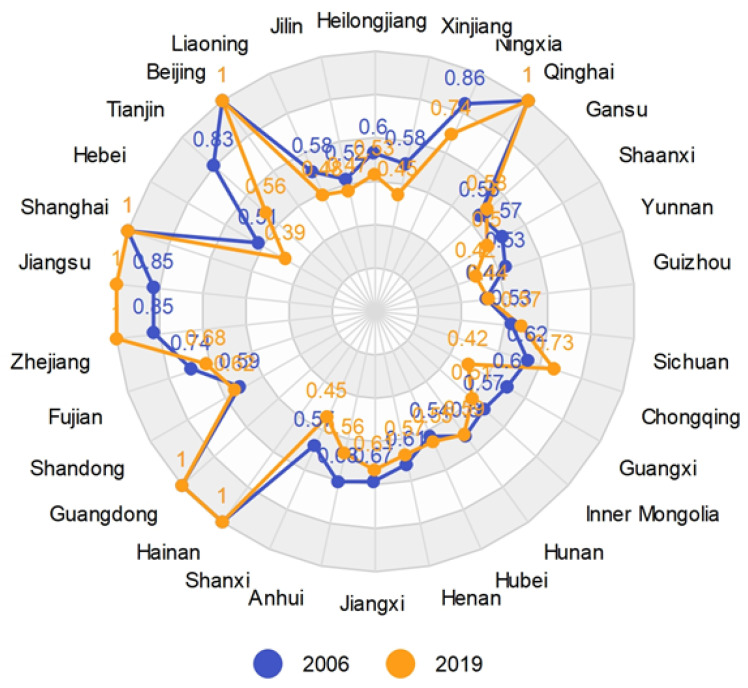
China’s carbon emission efficiency values by provinces in 2006 and 2019.

As can be seen from Figure 2, the carbon emission efficiency from 2006 to 2019 shows that the eastern region is the most efficient, the western region is the second most efficient, the central region is more backward, and the northeast region is the least efficient. Further calculations show that the average value of efficiency in the eastern region reaches 0.818, while 0.543, 0.581, and 0.471 in the central, western, and northeastern regions, respectively. From the perspective of provincial efficiency, it can be seen that Beijing, Shanghai, Guangdong, and Hainan, which belong to the eastern region, have carbon emission efficiency values of 1.000 in each year from 2006 to 2019. Exploring reasons for this, we can find that Beijing and Shanghai attach importance to sustainable economic development and have been implementing strict environmental regulation policies. Moreover, these two are ahead of other regions in China in terms of investment and technology in environmental protection, which have significant effects on low-carbon development. Although Guangdong has a large population, it has a technological advantage over most provinces. Hainan is a large tourism province with a low share of secondary industry. Qinghai has a small population, and it is also at the forefront of production, which is effective for the DMU. In addition, driven by the radiation effect of Shanghai, the carbon emission efficiency of Jiangsu and Zhejiang reach the production frontier in some years. Many provinces with relatively poor performance in carbon emission efficiency are primarily due to a less than optimal energy structure with extensive production, and most of these economies are driven by industrial development. In particular, as an old industrial base in China, the transformation process in Northeast China is limited by its geographical location and resource conditions. Overall, the carbon emission efficiency value in China is 0.642, indicating that there is still more room for improvement in carbon emission management. As shown in the carbon emission efficiency radar charts of provinces in 2006 and 2019 (Figure 3), although more provinces reached carbon emission efficiency in 2019, the regional differences are still obvious and even tend to widen.

### 4.2. Analysis of Regression Results of the Second Stage Stochastic Frontier Model

The three slack variables selected in this paper are capital stock, labor force and total energy consumption. Through the summary and generalization of related literature, we select external drivers such as the intensity of finance in environmental protection, level of economic development, industrial structure, level of urbanization, degree of openness and level of scientific and technological innovation as independent variables in the stochastic frontier model. The results are shown in Table 3.

According to the regression results, we compared the one-sided generalized likelihood ratio test results with the critical values. We found that they conformed to the characteristic of chi-square distribution, so the table of critical values adopted chi-square distribution table. The six external driver variables indicated that the degree of freedom is 6, and the critical value is 16.812 at 1% level of significance. It is clear that all the input variables reject the original hypothesis that there is no inefficiency under the 1% level of significance, so it is reasonable to use the stochastic frontier model.

In addition, gamma is between (0,1), indicating the existence of external drivers and random noise interfering with the emission efficiency values of each dimension, so the original input needs to be adjusted. A positive correlation coefficient indicates that an increase in this environmental variable is not conducive to efficiency and can result in input waste, while a negative one is beneficial to efficiency.

Table 3 shows that the six external drivers have significant effects on the slack variables, among which the intensity of finance in environmental protection and the degree of openness have negative effects. The increase in local government spending on environmental protection can reduce input redundancy, which is significant for improving the overall efficiency of carbon emissions. The increase in the degree of openness, which introduces advanced technology and equipment, has similar effects. The level of urbanization and industrial structure have positive effects on the three slack variables, and the increase in the level of urbanization and the development of the secondary industry are not conducive to the improvement of carbon emission efficiency. Economic development will consume capital stock, but will also drive employment and increase energy consumption. In terms of the scientific and technological innovation level, the capital stock is in 100 million yuan, and the other two input indicators are in millions of people and million tons of standard coal as units. The coefficient of science and innovation level on the capital stock is −29.370, and the coefficients of the other two input indicators are 0.227 and 4.440. We found that the impact of innovation level on the total input is negative after the calculation of uniform unit summing, thus the innovation will help the improvement of carbon emission efficiency.

### 4.3. Analysis of the Revised Results of Carbon Emission Efficiency of Each Province under the Third-Stage SBM-Undesirable Model

**Figure 4:**
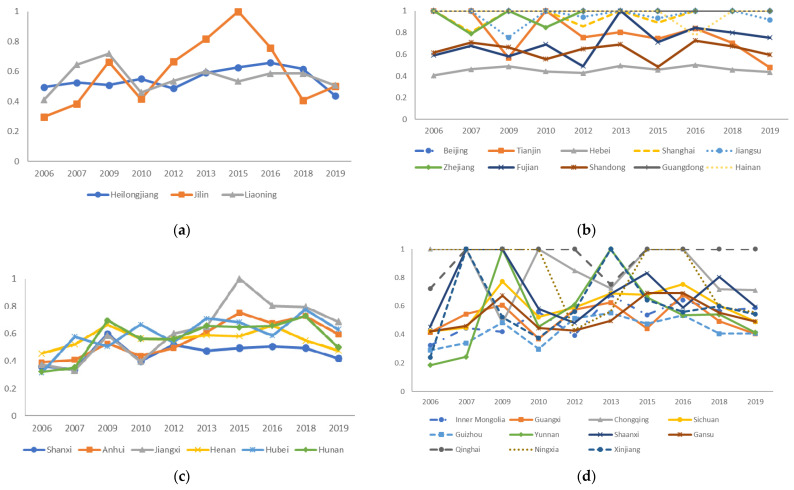
(**a**) Revised results of carbon emission efficiency in Northeastern China, 2006–2019; (**b**) revised results of carbon emission efficiency in Eastern China, 2006–2019; (**c**) revised results of carbon emission efficiency in Central China, 2006–2019; (**d**) revised results of carbon emission efficiency in Western China, 2006–2019. Note: To ensure the effect of the figure, the results of 2008, 2011, 2014, and 2017 are not shown; please contact the author to obtain them if necessary.

Based on the adjusted input and the original output in the second stage, the efficiency values of each DMU are remeasured using the SBM model considering undesirable outputs, and the results are shown in Figure 4.

As can be seen from Figure 4, the revised average value of carbon emission efficiency of four major economic regions in China from 2006 to 2019 has increased compared with the first stage. From the perspective of provincial efficiency, Beijing and Guangdong still reach the effective frontier with the carbon emission efficiency values of 1.000 for each year from 2006 to 2019. Heilongjiang, Tianjin, Shanghai, Jiangsu, Zhejiang, Hainan, Shanxi, Anhui, Jiangxi, Inner Mongolia Autonomous Region, Guizhou, Gansu, Qinghai, and Ningxia Hui Autonomous Region have slightly lower efficiency values in the third stage compared with the first stage, indicating that their efficiency values are overestimated due to the influence of environmental variables. While the adjusted carbon emission efficiency of the remaining provinces has improved to some extent. Further calculation gives that the northeast region has improved by 17.82% compared with that at the first stage, which indicates that its previous efficiency was influenced by external adverse environmental factors and stochastic noise, making the effect of management on carbon emission efficiency exaggerated.

The overall carbon emission efficiency values in China are shown in Table 4. In this paper, we calculate the values of Q1, Q2 and Q3 quartiles, which can clearly show that both in quantile and mean values, the corrected efficiency has improved over the initial efficiency, indicating that environmental factors have a significant impact on carbon emission efficiency.

**Figure 5:**
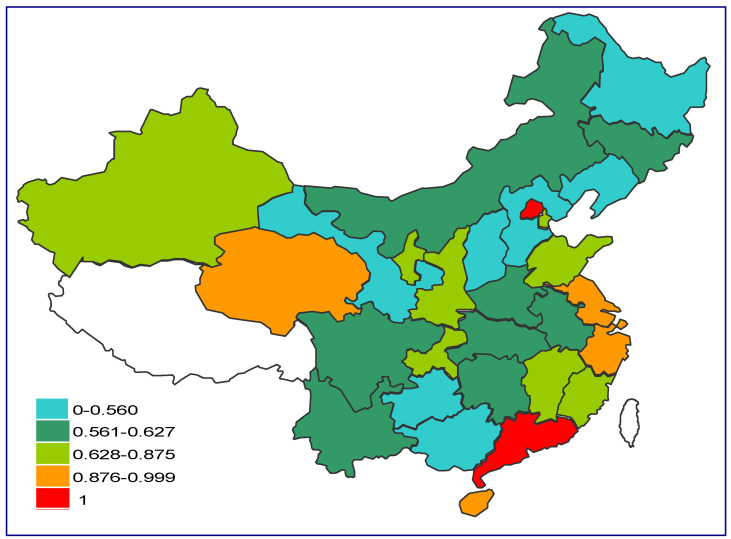
Distribution of the mean value of the revised carbon emission efficiency results by province in China, 2006–2019.

Based on the results of the three-stage revised efficiency and combined with the quartile results, we plot the mean classification distribution of the revised carbon emission efficiency of each province in China. As shown in Figure 5, the carbon emission efficiency of the eastern region is significantly higher than that of other regions, indicating that the carbon emission management in this region is relatively good compared to that in other regions. Combined with Table 4 and Figure 4, it can be seen that carbon emission efficiency in the northeastern, central and western regions is lower than the average level in China, while the overall efficiency of Northeast China is lower than that of Central China and West China. The industrial structure of Northeast China is mostly industry or even heavy industry, and its transformation into the tertiary industry or other types will be a long-term process. The way to effectively improve efficiency in this region still lies in technology and management, and the more inefficient the region is, the more attention should be paid to the improvement of technology and carbon emission management. In addition, it can be more intuitively seen that Beijing, Guangdong, Shanghai, Jiangsu, and Zhejiang, located in the eastern coastal region, are carbon emission efficient regions, and Qinghai in the central region also performs well.

### 4.4. Forecast of Inter-Provincial Carbon Emission Efficiency Values Based on LSTM Model in the Next Five Years

**Figure 6:**
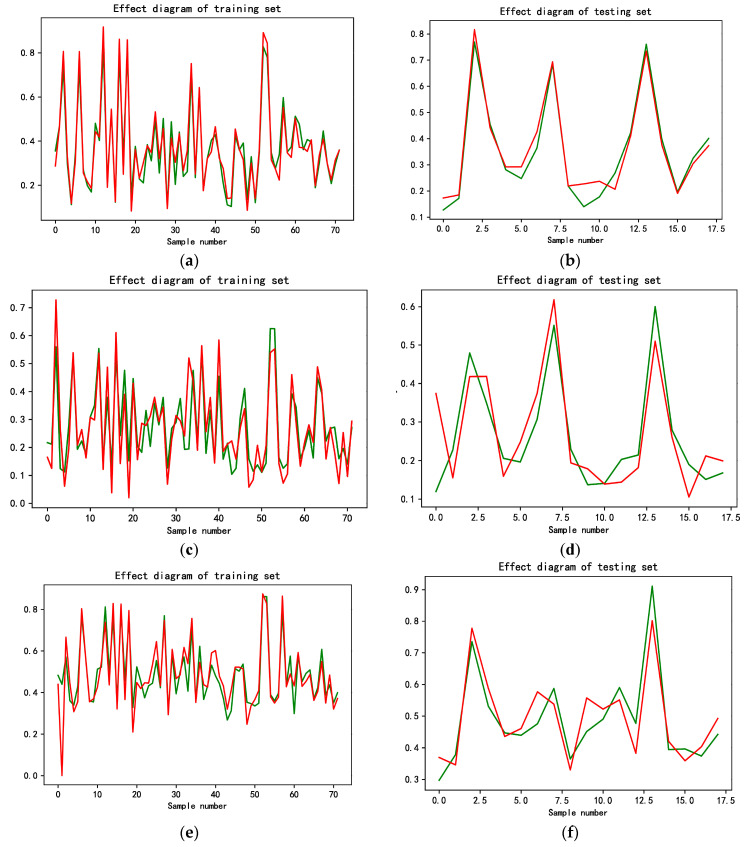

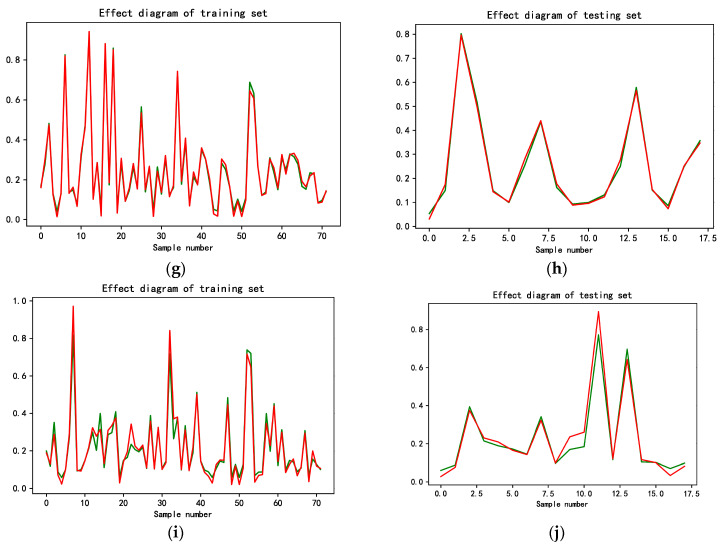
(**a**) Fitting effect diagram of labor force training set; (**b**) fitting effect diagram of labor force testing set; (**c**) fitting effect diagram of capital stock training set; (**d**) fitting effect diagram of capital stock testing set; (**e**) fitting effect diagram of total energy consumption training set; (**f**) fitting effect diagram of total energy consumption testing set; (**g**) fitting effect diagram of gross regional product training set; (**h**) fitting effect diagram of gross regional product testing set; (**i**) fitting effect diagram of carbon dioxide emissions training set; (**j**) fitting effect diagram of carbon dioxide emissions testing set.

We first conceived a direct time series prediction by a single variable (carbon emission efficiency value). Since this efficiency is weighted by multi-input and multi-output indicators, and external environmental factors are excluded. So, the data lack smoothness, and the fitting effect of the training set is poor, which is not enough to produce a reliable prediction result. However, we observed the original multi-input and multi-output data, and found that most of the original indicators are consistent with the smoothness characteristics. Therefore, we make the forecast of each input and output indicator in the next five years based on the LSTM model. We also notice the efficiency values are in the interval of [0, 1], so no normalization pre-processing of the data is needed, and the prediction results are not subject to inverse normalization. However, the indicators need to be normalized and inverse normalized, since these indicators do not have characteristics like the data of carbon emission efficiency. The total number of observed provinces is 30 and the sample size is small, so we use a sliding time window to expand the sample set, forming three groups of samples from 2006 to 2016, 2007 to 2017, and 2008 to 2018. We also fit the indicator of the last year in each group and form a total of 90 samples, with 80% of the samples as the training set and 20% as the test set. In this paper, the prediction effect of the LSTM model is judged based on R^2^, MSE, and MAE. To ensure the stability of the results, we make nine measurements for all indicators. The R^2^ is sorted from largest to smallest, and then we take the median. The corresponding results are shown in Table 5, and the fitting effect of the training set and test set is plotted in Figure 6. According to the results, most of the indicators show a good fitting effect and there is no overfitting, which has a certain generalization ability and provides a guarantee for the reliability of prediction.

**Figure 7:**
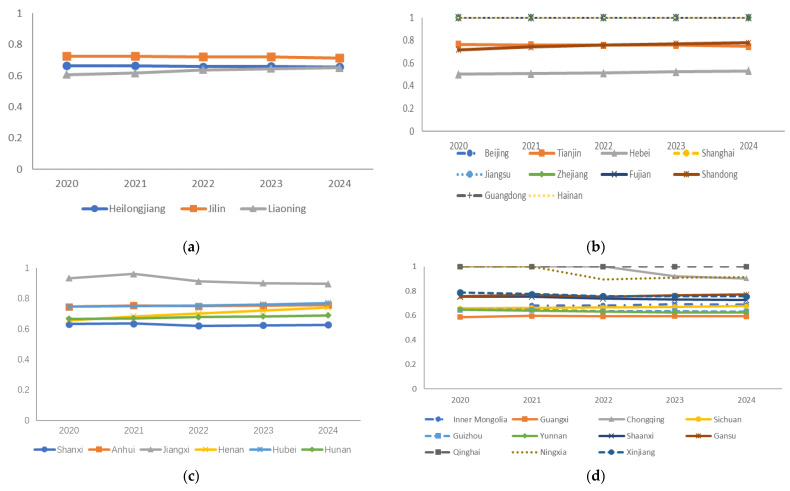
(**a**) Projected results of carbon emission efficiency in Northeastern China in the next five years; (**b**) projected results of carbon emission efficiency in Eastern China in the next five years; (**c**) projected results of carbon emission efficiency in Central China in the next five years; (**d**) projected results of carbon emission efficiency in Western China in the next five years.

On the basis of the predicted data and the SBM-undesirable model, we measured the future efficiency values of 30 provinces. Since the index data prediction is based on the sample data after excluding external environmental factors, only the third-stage remeasurement is needed. The results are shown in Figure 7.

## 5. Main Conclusions and Recommendations

### 5.1. Main Conclusions

The main conclusions of this paper are as follows: (1) From the static analysis results, we know that the average value of carbon emission efficiency in China from 2006 to 2019 is 0.642 in the first stage and 0.696 in the third stage, which are ineffective, and there is much room to improve. From the provincial perspective, five provinces reach the effective carbon emission before the adjustment, namely, Beijing, Shanghai, Guangdong, Hainan, Qinghai. After the adjustment, Shanghai, Hainan and Qinghai are no longer in the effective frontier, and their average efficiency value has decreased. There are 12 provinces with higher efficiency values than the average after the adjustment, one more than before the adjustment. From the perspective of the four major economic regions, the adjusted carbon emission efficiency ranking is Eastern Region > Western Region > Central Region > Northeast Region, and only the eastern region has an efficiency value higher than the average.

(2) From the perspective of dynamic analysis, from 2006 to 2019, the carbon emission efficiency values of Beijing and Guangdong before and after adjustment are still maintained at 1 every year, reaching the effective frontier. In the third stage, the influence of external drivers and random noise is removed, so that all DMUs are adjusted to the same external environment, at which time Shanghai, Hainan and Qinghai no longer maintain the carbon emission efficiency of 1 in the first stage. According to the analysis of variance, the average annual fluctuation of carbon emission efficiency in China becomes smaller after the adjustment, and there are large differences between provincial areas.

(3) Based on the results of SFA regression analysis, it can be seen that the six external drivers selected in this paper have significant effects on the input slack variables, so it is necessary to eliminate the effects of external drivers. Among them, the intensity of finance in environmental protection and the degree of openness have negative effects on the slack variables, and the enhancement of these indicators is favorable to the improvement of carbon emission efficiency. The increases in urbanization level and the secondary industry have positive effects on the slack variables, which are not beneficial to the improvement of carbon emission efficiency. The economic development will consume the capital stock but promote employment, as well as increase energy consumption. The impact of science and technology innovation level on the total input slack variables is negative.

(4) We analyzed the annual average carbon emission efficiency values of each province under the three-stage SBM-undesirable model, and classified them into five levels. Beijing and Guangdong are effective regions. Jiangsu, Zhejiang, Shanghai, Hainan and Qinghai are less effective regions. Gansu, Liaoning, Heilongjiang, Guangxi Zhuang Autonomous Region, Shanxi, Guizhou and Hebei are inefficient regions. Sichuan, Hubei, Yunnan, Anhui, Hunan, Henan, Jilin, and Inner Mongolia Autonomous Region are the medium efficient regions, and the remaining provinces are the medium- high efficient regions.

(5) The prediction results of carbon emission efficiency values based on the LSTM model show that eight provinces will reach effective carbon emission in the next five years, namely, Beijing, Shanghai, Jiangsu, Zhejiang, Fujian, Guangdong, Hainan, and Qinghai, but the gap between provinces still exists. In addition, the overall national carbon emission efficiency is gradually improving, and the values of all regions will also improve, with the greatest improvement in the central region.

### 5.2. Recommendations

This paper proposed the following suggestions based on the results of efficiency value and predicted efficiency value combining the regression of six external-driven factors.

(1) Carbon emission efficiency in China is highly differentiated across regions, and there are still disparities among provinces after five years of prediction. Additionally, the adjusted carbon emission efficiency ranking is Eastern Region > Western Region > Central Region > Northeast Region. Therefore, it is essential to develop distinctive strategic policies that take advantage of the region’s own strengths. Specifically for the four major economic regions, the eastern region has the highest carbon emission efficiency and exceeds the average level in China. Hence, the eastern region should take advantage of its own strengths, continue to maintain a high-quality level of economic development, and promote the steady improvement of carbon emission efficiency. The western region should pay more attention to the adoption of technology, strengthen the construction of urbanization, and form an urban development path with ecological livability as the basic feature. The central region should increase local financial support for environmental protection, promote the construction of clean energy centers, and promote low-carbon urban development. The northeast region, as the region with the lowest carbon emission efficiency, should focus on reducing high energy-consuming industries, promoting the transformation of regional industrial structure by importing advanced technologies and talents, and enhancing the quality and efficiency of industries through upgrading technologies and equipment.
(2) We should focus on the structural layout of the industry and develop new business models driven by technological innovation. In recent years, China still has the problem of unreasonable industrial structure and overcapacity. Based on the results of this paper, it can be seen that the percentage of secondary industry has a negative impact on the carbon emission efficiency level, especially in Heilongjiang and Hebei, where the secondary industry is the predominant industry, the carbon emission efficiency value is relatively low. Therefore, similar provinces should optimize their industrial structure, emphasize technological innovation, and upgrade their carbon emission technologies. The government should integrate the three major industries to reduce carbon dioxide emissions by focusing on the development of strategic new industries in order to reduce industrial energy consumption and drive the development of new business models. In this way, it will promote the level of carbon emission efficiency and realize the sustainable path of low-carbon economic development.
(3) From the empirical results in the paper, it can be seen that opening up to the outside world has a positive effect on carbon emission efficiency. Beijing, Shanghai, Jiangsu, Zhejiang, Fujian, Guangdong, and Hainan are currently at a high level of efficiency. At the same time, all of these regions are at a high level of openness to the outside world and will achieve effective carbon emission efficiency values in the next five years. Therefore, provinces should be aware of the characteristics of the development of foreign cities similar to their own provinces and absorb advanced foreign technologies by recognizing these characteristics. The provincial situation should be considered to open the road to the outside world and form an effective docking with the outside, not only the cooperation and exchange of foreign regions, but also the coordinated development of other domestic provinces. In this way, we can form a low-carbon development of internal and external circulation channel to promote low-carbon development. Ultimately, by optimizing the opening structure, the internal and external cycles of low-carbon development can be realized.

## Figures and Tables

**Table 1:** Carbon emission efficiency evaluation index system.

Input Indicator	Input–Output Indicator	Indicator Interpretation	Data Source
Input indicator 1	Labor force	Number of employed people in each region at the end of the year, unit: 10,000 people	China Statistical Yearbook by Province
Input indicator 2	Capital stock ^1^	Capital stock as capital input, unit: 100 million yuan	China National Statistical Yearbook
Input indicators 3	Total energy consumption	Total energy consumption by region, unit: million tons of standard coal	China Energy Statistical Yearbook
Expected output indicator	Gross regional product	Real GDP of each region, unit: 100 million yuan	China Statistical Yearbook, deflated by 2005 as the base period
Undesirable output indicator	Carbon dioxide emissions	Carbon dioxide emissions by region, units: ton	China Carbon Accounting Database

^1^ Capital stock is calculated through the amount of capital in the base period, the selection of depreciation rate and current investment indicators, and then the total investment is deflated in fixed assets of the whole society [35].

**Table 3:** Regression results of stochastic frontier model.

	Capital Stock Slack Variable	Labor Slack Variable	Total Energy Consumption Slack Variable
Constant	−17,189.032 ***	898.620 ***	−6,336.820 ***
Intensity of finance in environmental protection	−618,624.470 ***	−110,921.630 ***	−402,584.220 ***
Level of Economic development	0.152 ***	−0.004 **	−0.026
Industrial structure	9,875.560 ***	236.204	7,083.186 ***
Level of urbanization	32,034.435 ***	816.628 **	22,288.015 ***
Level of openness	−0.847 ***	−0.078 ***	−0.808 ***
Level of scientific and technological innovation	−29.370 **	0.227	4.440 ***
Sigma-squared	142,929,060.000	1,398,628.300	58,676,838.000
gamma	0.748	0.950	0.853
LR	233.065 ***	700.140 ***	388.725 ***

Note: *** and ** represent passing the test of significance level 1% and 5%, respectively.

**Table 4:** Comparison of the overall distribution of initial and modified efficiencies.

	Initial Efficiency	Corrected Efficiency
Mean value	0.642	0.696
Q1	0.489	0.561
Q2	0.547	0.628
Q3	0.823	0.876

**Table 5:** Evaluation index data of the fitting effect of each input–output index.

	Training Set R^2^	Training Set MSE	Training Set MAE	Testing Set R^2^	Testing Set MSE	Testing Set MAE
Labor force	0.939	0.002	0.036	0.959	0.002	0.032
Capital stock	0.707	0.006	0.057	0.654	0.007	0.064
Total energy consumption	0.724	0.006	0.052	0.819	0.004	0.052
Gross regional product	0.994	0.000	0.013	0.995	0.000	0.011
Carbon dioxide emissions	0.946	0.002	0.027	0.955	0.002	0.029

## Data Availability

The data of this study can be obtained by contacting the author, email address: zhangzs0216@163.com.

## Abbreviations

Abbreviation	Definition
ANNs	Artificial Neural Networks
BCC	Banker, Charnes and Cooper
CCR	Charnes, Cooper and Rhodes
CO2	Carbon Dioxide
DEA	Data Envelopment Analysis
DMU	Decision-Making Unit
GARCH	Generalized AutoRegressive Conditional Heteroskedasticity
GDP	Gross Domestic Product
LR	Likelihood Ratio
LSTM	Long Short-Term Memory
MAE	Mean Absolute Error
MSE	Mean Square Error
R2	Coefficient of Determination
RNN	Recurrent Neural Network
SBM	Slack Based Measure
SFA	Stochastic Frontier Analysis
VAR	Vector Autoregression
